# Supplementary material for: Fabrication of Radiopaque, Drug-Loaded Resorbable Polymer for Medical Device Development
Source: Polymers (Basel). 2025 Mar 7;17(6):716. doi: 10.3390/polym17060716 (PMC11945502; doi:10.3390/polym17060716)
Supplement: Supplementary file 1 [file polymers-17-00716-s001.zip › polymers-3492793-supplementary.pdf]

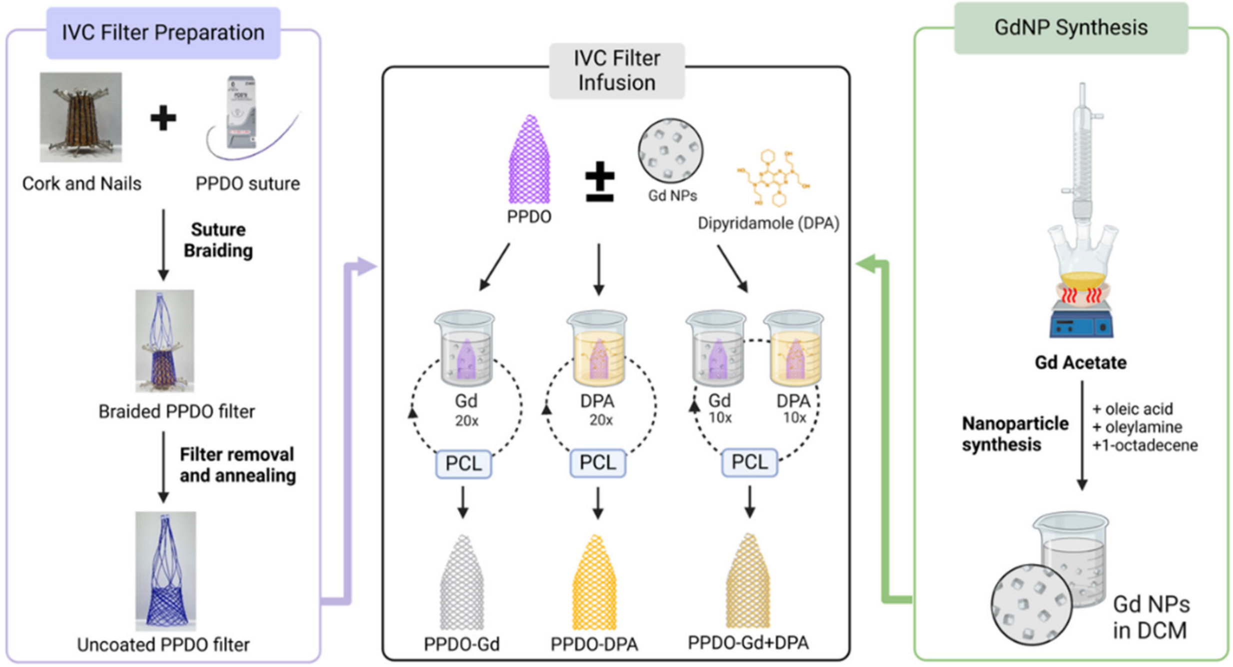

Figure S1. Schema for the fabrication of GdNP, DPA, and GdNP+DPA loaded IVCFs. The method involves braiding the poly(p-dioxanone) (PPDO) suture using cork and nails as the framework. Gadolinium nanoparticles (GdNPs) were synthesized via thermal decomposition of gadolinium acetate with oleic acid and dissolved in dichloromethane (DCM). The inferior vena cava filter (IVCF) was immersed in the nanoparticle solution, with or without dipyridamole (DPA), using the wet dipping technique. The polymer matrix relaxed in the presence of the solvent, allowing the nanoparticles and drug to permeate. Upon solvent evaporation, the nanoparticles and drug remain embedded in the polymer as it returns to its original form. Four IVCFs were prepared: control (PPDO only), PPDO-Gd, PPDO-DPA, and PPDO-Gd+DPA.

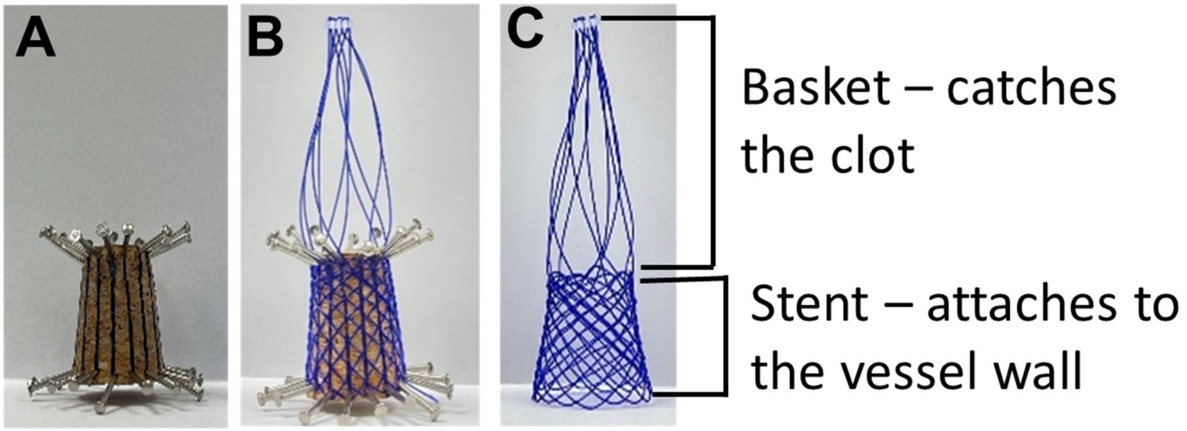

Figure S2. Fabrication process of the IVCF. (A) Nails were evenly spaced and secured to a cork as a guide for braiding. (B) Poly-p-dioxanone (PPDO) sutures were braided around the nails and a 3D-printed plastic tip to form the inferior vena cava filter (IVCF) structure. (C) After braiding, the nails were carefully removed, leaving behind the braided IVCF. The IVCF consists of two main components: the basket, which captures clots, and the stents, which anchor the device to the vessel wall for stability and function.
